# ^137^Cesium Exposure and Spirometry Measures in Ukrainian Children Affected by the Chernobyl Nuclear Incident

**DOI:** 10.1289/ehp.0901412

**Published:** 2010-01-25

**Authors:** Erik R. Svendsen, Igor E. Kolpakov, Yevgenia I. Stepanova, Vitaliy Y. Vdovenko, Maryna V. Naboka, Timothy A. Mousseau, Lawrence C. Mohr, David G. Hoel, Wilfried J.J. Karmaus

**Affiliations:** 1 University of South Carolina, Columbia, South Carolina, USA; 2 Research Center for Radiation Medicine, Academy of Medical Sciences of Ukraine, Kyiv, Ukraine; 3 Radioecological Center, Ukrainian National Academy of Sciences, Kyiv, Ukraine; 4 Medical University of South Carolina, Charleston, South Carolina, USA

**Keywords:** ^137^cesium, Chernobyl, children, environmental, epidemiology, ionizing radiation, pulmonary, spirometry

## Abstract

**Background:**

After the Chernobyl accident in 1986, children of the contaminated Narodichesky region of Ukraine were obliged to participate in a yearly medical screening. They have been exposed to ^137^cesium (^137^Cs; half-life = 30 years) in contaminated soils, air, and food.

**Objective:**

Using a “natural experiment” approach and a longitudinal prospective cohort study design, we investigated the association of soil ^137^Cs and spirometry measures for 415 children using 1,888 repeated measurements from 1993 to 1998.

**Methods:**

Mean baseline village soil ^137^Cs measurements, which varied from 29.0 to 879 kBq/m^2^, were used as exposure indicators. A standardized spirometry protocol and prediction equations specific to Ukrainian children were used by the same pulmonologist in all screenings.

**Results:**

Children living in villages with the highest quintile of soil ^137^Cs were 2.60 times more likely to have forced vital capacity (FVC) < 80% of predicted [95% confidence interval (CI), 1.07–6.34] and 5.08 times more likely to have a ratio of forced expiratory volume in 1 sec (FEV_1_) to FVC% < 80% (95% CI, 1.02–25.19). We found statistically significant evidence of both airway obstruction (FEV_1_/FVC%, peak expiratory flow, and maximum expiratory flow at 25%, 50%, and 75% of FVC) and restriction (FVC) with increasing soil ^137^Cs.

**Conclusions:**

These findings are unique and suggest significant airway obstruction and restriction consequences for children chronically exposed to low-dose radioactive contaminants such as those found downwind of the Chernobyl Nuclear Power Plant.

The long-term health and environmental consequences of the Chernobyl catastrophe are not yet fully reported despite 23 years of research (Moller and Mousseau 2006; Zakharov and Krysanov 1996). There is considerable disagreement among government agencies, health professionals, and scientists over the long-term effects of radiologic contaminants (Mousseau et al. 2005; Zakharov and Krysanov 1996), and the official position of the United Nations (UN) [Balanov 2005; International Atomic Energy Association (IAEA) 2005, 2006; World Health Organization (WHO) 2006] has been generally interpreted to suggest that the consequences to human health are much lower than expected (Anspaugh 2007; Geras’kin et al. 2008; Yablokov 2009a), although the authors of the UN reports concluded that “given the lack of statistical power based upon the estimated doses and confounding variables from causes other than radiation exposure, studies of the causes of mortality of the general population or evacuees from highly contaminated zones are unlikely to provide useful scientific information on radiation effects” (WHO 2006). This position essentially negates any possibility of conducting useful epidemiologic studies without having individual-based dose measurements, which is a near impossibility for the populations most affected by Chernobyl fallout. Similarly, the parklike appearance of the 2044.4-km^2^ Chernobyl exclusion zone, with some animals appearing to be increasing in numbers, suggests an ecosystem on the rebound, and the UN position (IAEA 2006) has been that there is little evidence to suggest significant large and persistent effects of contamination on the biota of the zone. However, the UN reports, and interpretations of it in the popular and scientific press, have generated an optimism that may be based on too few studies published in English, conducted too soon after the event to be conclusive. A growing number of reports in the literature suggest measurable effects for both human and animal populations. Recently, a thorough literature review was published on this subject (Yablokov 2009a, 2009b; Yablokov and Nesterenko 2009; Yablokov et al. 2009a, 2009b). A prudent approach to this problem should involve rigorous scientific explorations of all available information.

Several β- and γ-emitting radionuclides were released from the Chernobyl nuclear reactor, including ^131^iodine, and ^137^cesium (^137^Cs). ^131^Iodine has a half-life of 8 days, and ^137^Cs, 30 years. Most of the acute health effects from the Chernobyl incident have been compared with ^131^iodine exposures, whereas ^137^Cs has been the radioisotope of greatest concern for persistent exposures in the affected communities. In many rural villages across the Ukraine and Belarus, people are routinely exposed to ^137^Cs through their diet of locally grown food that bioaccumulates ^137^Cs and use of drinking water derived from shallow, open wells (Nesterenko et al. 2009a; Yablokov et al. 2009a, 2009b). In addition, forest fires and agricultural activities in the contaminated regions of Ukraine and Belarus also release radionuclides to the atmosphere as aerosols or attached to dust particles, which are then widely transported (Konoplia et al. 1992) and are potentially inhaled in adjacent regions. Recent summaries of Belarusian and Ukrainian reporting agencies suggest that ingestion is a significant source of exposure for large segments of these populations (Nesterenko et al. 2009a, 2009b; Yablokov and Nesterenko 2009).

The short-term effects on the lung and pulmonary system after acute high-dose exposure to radiation are relatively well known, and long-term effects are occasionally reported (Abid et al. 2001; Coggle et al. 1986; Yablokov 2009b). However, comparatively little research has assessed the long-term health effects of persistent ^137^Cs or other radioisotope exposures on the lung and pulmonary system (Yablokov 2009b). There is increasing evidence that long-term exposure to radioisotopes such as ^137^Cs is associated with modulation of the immune system (Yablokov 2009b). Such modulation of immune function may lead to recurrent infection, and the most commonly reported long-term effect on the pulmonary system from persistent exposure to ^137^Cs or other radioisotopes is increased pulmonary infection (Yablokov 2009b). Detrimental functional effects of recurrent pulmonary infection during childhood lung development have been suggested (Gern et al. 2005). However, we are unaware of any study reported in English that has documented deficits in lung function or increased bronchial reactivity in children associated with chronic exposure to ^137^Cs or other radioisotopes.

We studied the association of average soil ^137^Cs levels in a community with pulmonary function and reactivity measures of children living in this community to test the hypothesis that prolonged exposure to ^137^Cs during childhood development reduces lung function and increases airway reactivity. To test this hypothesis, we conducted an epidemiologic study using a “natural experiment” design that took advantage of the highly heterogeneous nature of contaminant deposition during the Chernobyl radiologic incident. Because of variation in wind direction and rainfall during the nuclear fire that burned for 10 days starting 26 April 1986, and variation in local soil types, contaminant levels can vary by two orders of magnitude between villages within a small region. This provides a unique opportunity to conduct relatively controlled comparisons among groups of individuals who share most elements of their living and socioeconomic conditions but who have been chronically exposed to widely different levels of contaminants.

We used a prospective longitudinal cohort study design with data from a population of children under annual medical surveillance who were currently exposed to ^137^Cs through their drinking water and diet of locally grown food in the Narodichesky region of Ukraine, a region adjacent to an evacuated region heavily contaminated by radioactive fallout from the 1986 Chernobyl radiologic incident.

## Methods

### Study population

The Narodichesky district is a farming area of Ukraine about 80 km west of the Chernobyl Nuclear Power Plant. Since the Chernobyl radiologic incident in 1986, the district has become quite poor. This area experienced considerable fallout from the Chernobyl incident, and soil contamination levels for ^137^Cs from the 38 Narodichesky municipal areas where families with children still live vary from 29 to 879 kBq/m^2^ (typical natural background levels in the United States from radon are about 20 kBq/m^2^) (Mossman 2007). Every year since December 1986, all children from the Narodichesky district of the Zhytomyr Oblast within Ukraine have been required by law to participate in a public health intervention that includes a yearly medical screening because they are exposed to ^137^Cs in their drinking water and diet due to their consumption of locally grown produce. We have named these medically screened children the Narodichi Children’s Cohort (NCC). NCC children were taken by bus to the central district hospital within the village of Narodichi for all health screenings. The goals of the Narodichesky public health intervention and associated medical screenings have always been to identify and track the health state of the children each year with sensitive clinical measurements and to provide medical treatments to those in need.

Data from the NCC medical screenings during 1993–1998 were entered into electronic databases and were used exclusively in this study. All children who were assessed in 1998 were selected for inclusion in our analytical data set, and additional data for each child were entered into our database from the 1993–1997 medical screenings for those children. This NCC analytical data set has 1,459 children who were assessed up to six times for a total of 5,519 medical screenings. The NCC represents a dynamic cohort; children did not participate in all years and left the study when reaching 18 years of age or when their family moved away from the area. The population in this region has been stable during the childhood of this cohort, with only four children moving between villages during 1993–1998; those children were not included in our analysis. Additional information on the larger NCC study population is provided elsewhere (Stepanova et al. 2008).

### ^137^Cs assessment

Mean contamination levels of ^137^Cs were calculated from measurements taken from the soils surrounding the 38 Narodichesky villages (Likhtarev et al. 2002). Sample collection, analysis, correlation with individual dose, and spatial modeling of these data are summarized elsewhere (Stepanova et al. 2008). The mean village contamination level from the village where each child resided was used as our estimate of exposure to ^137^Cs. Another study has demonstrated that residential and individual ^137^Cs internal doses are highly correlated (Sharifov et al. 1996). Further details on use of these data to estimate exposure in the NCC are presented elsewhere (Stepanova et al. 2008).

### Outcome assessment

The NCC medical screening included a battery of tests and measurements to look at the breadth of potential health effects potentially associated with dietary exposure to ^137^Cs. These included spirometry and airway reactivity testing for every third child examined. All diagnoses were recorded using the *International Classification of Diseases, 9th Revision* (ICD-9; WHO 1975), and appropriate treatments were prescribed by the screening physician. Spirometry was performed using the same spirometer (Pneumotachograph-Automatic-01; Polytechnical University of Kiev and the Institute of Physiology, National Academy of Science of the Ukraine, Kyiv, Ukraine) and by the same pulmonologist (I.E.K.) in all assessments during these selected years using a standardized Ukrainian protocol (Chaialo et al. 1991; Stepanova et al. 2000) based on the European Respiratory Society guidelines (European Respiratory Society 1993; Kurch 1987; Rachinsky and Tatochenko 1988). We have reported all spirometry measures as the percentage of predicted values by using standardized prediction equations for Ukrainian children (Rachinsky and Tatochenko 1988; Shiryaev 1978). Furthermore, we have reported the following percentage of predicted spirometry measures: forced expiratory volume in 1 sec (FEV_1_), peak expiratory flow (PEF), forced vital capacity (FVC), Tiffeneau index (FEV_1_/FVC%, the ratio of FEV_1_ to FVC), and maximum expiratory flow at 25% (MEF_25_), 50% (MEF_50_), or 75% (MEF_75_) of the FVC. Lowest limit of normal equations were not available for Ukrainian children, so we looked at decreased spirometry measures by identifying tests where the measures were less than 80% of their predicted value, and generated binary variables to represent these decreased values. Airway reactivity was assessed by performing postbronchodilator (salbutamol; GlaxoSmithKline, Poznan, Poland) spirometry with all children who received spirometry testing (European Respiratory Society 1993). Children were considered to have airway reactivity if their MEF_25_, MEF_50_, or MEF_75_ values improved at least 10% after bronchodilator administration (defined as large, middle, and small airway reactivity, respectively).

### Spirometry subgroup

Children whose height was < 1 m at the time of the screening were not eligible for pulmonary function testing (*n* = 740, 13.4%). In the absence of their parents, children were asked about many general health risk factors of their parents’ behaviors (e.g., alcohol abuse, tobacco use). Children were also asked about their own tobacco use. All children were excluded from spirometry testing if they reported to the pulmonologist that they were tobacco smokers. In addition, we have limited our analyses to those children without current infections (e.g., pneumonia, common cold, bronchitis) at the time of measurement. Given these limitations, our final data set included 415 children assessed by 1,888 repeated measurements (Table 1). Our spirometry analytical subgroup was selected as follows: all children who received a medical screening (*n* = 1,459), minus four children who moved to a different village (*n* = 1,455), minus 84 children diagnosed with a current infection (*n* = 1,371), and minus 956 children who were < 1 m in height at the time of screening or were not selected for spirometry (final *n* = 415).

### Data analysis

Data from each individual were analyzed with a prospective longitudinal cohort design where children were essentially matched to themselves over time using SAS software (version 9.1; SAS Institute Inc., Cary, NC, USA). We applied linear (Proc Mixed) and logistic (Proc Genmod) mixed-effects models to adjust for the repeated outcome measurements of the child, random effects of both the child and date tested, the potential in some children born before the Chernobyl accident for acute radiation exposure in addition to chronic exposure, and the interaction of time with soil ^137^Cs. Age, sex, and height were implicitly adjusted for in spirometry measure models through the use of percent predicted measures (Rachinsky and Tatochenko 1988; Shiryaev 1978). Parental smoking prevalence was very similar in all the villages throughout the Narodichesky district (Stepanova and Vdovenko, 2006). Therefore, we did not adjust for parental cigarette smoking because it was unlikely to have changed between 1993 and 1998 (if a child’s parent did or did not smoke in 1993, then in this culture they most likely retained that habit in 1994–1998). As with our previous report (Stepanova et al. 2008), we used the regular maximum-likelihood method of statistical estimation. In the linear mixed model, we used a Gaussian correlation structure, and in the logistic mixed model we used an autoregressive correlation structure to address the correlation of the repeated measures within each participant.

### Ethics

The goal of the NCC has always been to help the children living in the contaminated regions within Narodichesky, and to do so in an equitable and medically robust manner for all affected children. Therefore, the NCC has always been a mandatory and thorough public health examination, and not a human subjects research study under the principles of the Declaration of Helsinki. Only relatively recently has there been interest in mining these public health data for scientific purposes. Because soil ^137^Cs measurements for each Narodichesky village became available to our team, it has become possible to now perform “natural experiment” epidemiology studies using both the NCC outcome and soil ^137^Cs data. Therefore, although human subjects research review by an authorized institutional review board was not necessary to initiate the medical screenings of the NCC, these statistical analyses of the data obtained from the NCC were submitted to and have been approved by the institutional review boards of the Research Center for Radiation Medicine, Academy of Medical Sciences of Ukraine, and the University of South Carolina.

## Results

### Study population

From 1993 through 1998, 415 children from 29 Narodichesky villages who met all inclusion criteria for this study were screened up to six times, for a total of 1,888 repeated measurements. Most children participated in five of these six screening years [mean, 4.99; 95% confidence interval (CI), 3.97–6.00; mode = 5]. The overall participation of the eligible children in the screening was 76.6%, and we found no significant differences across exposure quintile for participation in the screenings (*t* = −0.3, *p* = 0.737) or spirometry testing (*t* = −0.96, *p* = 0.345). Because exposure was assigned at the village level, we could not achieve exactly 20% for each exposure quintile group. Table 1 presents the descriptive statistics of the medical screening population, including the age range, birth year, participation by screening year, and soil ^137^Cs quintiles. Data from the children born in 1986, the year of the Chernobyl accident, were analyzed separately. Most of the children in our data set were born after the 1986 Chernobyl accident (59.2%). There were essentially no ethnic minorities in this region, and 48.9% of the children in this study were boys. Few of these children were overweight, as demonstrated by the mean body mass index of 17.0 kg/m^2^. As expected because of the inclusion criteria, younger children were less likely to have received spirometry (*p* < 0.0001). We found no significant difference in sex between the children who received spirometry and those who did not (*p* > 0.95).

### Soil ^137^Cs concentrations

The soil ^137^Cs distribution within this study population varied from 59.0 to 364 kBq/m^2^ with an interquartile range (IQR) of 220 kBq/m^2^. Table 1 shows the distributuion of NCC participants according to exposure quintiles. A more detailed description of the village-level soil concentrations and a map representing the spatial distribution of soil ^137^Cs concentrations within the Narodichesky Region have been published previously (Stepanova et al. 2008).

### Most frequent diagnoses

Table 2 presents the 15 most commonly reported ICD-9 diagnoses. Of those, the two diagnoses most strongly associated with increasing soil ^137^Cs exposure were codes 281.9, “other deficiency anemias—unspecified,” and 785.6, “enlargement of lymph nodes,” in both univariate and multivariate models (*p* < 0.05).

### Spirometry outcomes

The mean percent predicted spirometry measures were low, all between 80.0% and 90.0% of their predicted value. However, FEV_1_/FVC ratio was within the range of normal (mean = 96.9%; 95% CI, 86.4–107). As expected, all spirometry measures were significantly correlated with each other (*p* < 0.0001), with PEF and MEF_25_ having the strongest correlations (Pearson *r* = 0.86), and FVC and MEF_50_ having the weakest (Pearson *r* = 0.31). PEF and FEV_1_ were significantly correlated but less strongly than anticipated (Pearson *r* = 0.41, Spearman *r* = 0.40). PEF and FEV_1_ appear to be measuring different aspects of pulmonary function in these children and are not analogous measures (Aggarwal et al. 2006). All spirometry measures were significantly higher in children born after the 1986 Chernobyl incident and improved significantly with increasing time since then (*p* < 0.0001). Similarly, the adverse effects of soil ^137^Cs on all spirometry measures were significantly reduced with increasing time since the accident (data not shown).

### Multivariate models

We adjusted all multivariate models for repeated outcome measurements, random effects of both the child and date tested, risk factor of acute exposure to radiation in children born before the Chernobyl accident, and interaction of time with soil ^137^Cs. An IQR increase in soil ^137^Cs (223 kBq/m^2^) was associated with a 34.1% (95% CI, 2.6–75.4%) increase in the incidence of “enlarged lymph nodes” and 44.7% (95% CI, 7.0–96.8%) increase in “other deficiency anemias—unspecified” in multivariate mixed-effects logistic regression models. None of the other most common diagnoses (Table 2) were significantly associated with soil ^137^Cs (*p* > 0.05).

We generated results from multivariate linear regression models of each percent of predicted spirometry measure across interquartile-range (223 kBq/m^2^) soil ^137^Cs levels. We found that an IQR increase in soil ^137^Cs was significantly associated with reductions for all percent predicted flow measures, with the greatest reduction found with small airway flow (MEF_75_ = −13.8%; 95% CI, −11.5% to −16.1%) (Table 3). Similarly, percent predicted FVC decreased with increasing soil ^137^Cs levels (−11.1%; 95% CI, −9.9% to −12.4%). However, FEV_1_/FVC% was unassociated with soil ^137^Cs (β = 0.68, *p* > 0.05). We found that the assumption of linearity was violated in the highest soil ^137^Cs quintile for all spirometry measures. Therefore, we calculated similar multivariate models of the soil ^137^Cs quintiles.

In general, spirometry measures decreased with increasing soil ^137^Cs up to the second highest quintile, which had the lowest measures (all significantly lower than in the lowest quintile, *p* < 0.05) but increased slightly in the highest exposure quintile (Table 3). However, although greater than in the second highest exposure quintile, the spirometry measures in the highest exposure quintile were significantly less than in the lowest quintile for FEV_1_/FVC%, FVC, MEF_25_, and MEF_75_ (*p* < 0.05). The > 20% predicted deficits with the second highest quintile of soil ^137^Cs with PEF, MEF_25_, MEF_50_, and MEF_75_ suggest clinically relevant airway obstruction. Similarly, the deficits with MEF_75_ of > 20% predicted in the highest three exposure quintiles suggest that the small airways are most obstructed with increasing soil ^137^Cs. Although not always clinically significant, we found statistically significant evidence of both airway obstruction (FEV_1_/FVC%, PEF, MEF_25_, MEF_50_, and MEF_75_) and restriction (FVC) with increasing soil ^137^Cs.

We then looked at decreased spirometry measures, as defined by a measure being < 80% of the predicted measure. An IQR increase in soil ^137^Cs was associated with 3.86 times higher odds of having decreased FVC [odds ratio (OR) = 3.86; 95% CI, 1.99–7.48]. Regarding a decreased PEF, a per-IQR increase in soil ^137^Cs was associated with increased odds of having decreased PEF (OR = 2.33; 95% CI, 1.41–3.86). Decreased FEV_1_/FVC% (< 80% of FVC) was unassociated with a per-IQR increase in soil ^137^Cs (*p* > 0.10). The odds of having restrictive ventilator impairment (defined as FVC < 80% predicted and FEV_1_/FVC > 90% predicted) increased nearly three times with an IQR increase in soil ^137^Cs (OR = 2.97; 95% CI, 1.43–6.20). Airway hyperreactivity increased significantly with soil ^137^Cs only in the small airways (defined as MEF_75_ improved ≥ 10% after bronchodilator administration: OR = 1.76; 95% CI, 1.00–3.12). As with the adjusted mean models, we calculated similar multivariate logistic models with the soil ^137^Cs quintiles to assess the assumption of linearity (Table 4). Evidence of airway obstruction (decreased PEF and FEV_1_/FVC%) and restriction (decreased FVC) increased with increasing quintiles of soil ^137^Cs. Children living in villages with the second highest soil ^137^Cs exposure quintile were 6.07 times more likely to have their PEF < 80% predicted (95% CI, 2.09–17.62) and 5.83 times more likely to have small airway hyperreactivity (95% CI, 1.75–19.43) than were children living in villages with the lowest quintile of soil ^137^Cs. Similarly, children living in villages with the highest quintile of soil ^137^Cs were 2.60 times more likely to have their FVC < 80% predicted (95% CI, 1.07–6.34) and 5.08 times more likely to have their FEV_1_/FVC% < 80% (95% CI, 1.02–25.19). We found no significant differences between sexes in any of the spirometry measures associated with soil ^137^Cs.

To inspect which concurrent diagnoses were more predictive of spirometry decrements, we analyzed associations between diagnoses and lung function measures. Many children had suppressed immune systems: 14.7% prevalence of “other deficiency anemias—unspecified” (ICD-9 code 281.9), an indicator of suppression of myelopoiesis. Also, we found a 6.50% prevalence of “acute nasopharyngitis (common cold)” (ICD-9 code 460), which was the 15th most commonly diagnosed condition. Having a diagnosis of “other deficiency anemias—unspecified” was significantly associated with the concurrent spirometry results of FVC < 80%, PEF < 80%, and FEV_1_/FVC% < 90% (*p* < 0.0001) and was more strongly associated with decreased spirometry measures than any other diagnosis. Although pneumonia and bronchitis were not among the top 15 reported diagnoses in the NCC children, having such a diagnosis was strongly associated with having had a concurrent diagnosis of “other deficiency anemias—unspecified” (bronchitis: OR = 1.77; 95% CI, 1.01–3.11; pneumonia: OR = 4.37; 95% CI, 0.98–19.55). “Other deficiency anemias—unspecified” was the chronic illness diagnosis most significantly associated with both decreased spirometry (FVC and FEV_1_/FVC%) and ^137^Cs exposure (*p* < 0.0001).

## Discussion

We found that all spirometry measures decreased with increasing soil ^137^Cs, suggesting both obstructive and restrictive effects. The effects were most often strongest in the children living in villages with the second highest quintile of soil ^137^Cs, which is similar to what was previously found in this population with blood cell counts (Stepanova et al. 2008). These findings support the hypothesis that long-term exposure to ^137^Cs, likely through consumption of contaminated foods and local drinking water, is associated with decrements in lung function during child development.

Our study has some limitations. Soil ^137^Cs is not as precise as whole-body radiation dose measurements in estimating exposure to ^137^Cs. Despite this measurement error in our study, we found statistically significant associations between soil ^137^Cs and spirometry outcomes. Personal ^137^Cs dose data would help validate these findings and provide better information on the dosimetry of these effects. Also, we have screening data only from children who were well enough to travel for up to 120 min by bus to Narodichi for their health screening. The severely ill children within this community may not be included within our results, especially in the more remote villages, which would mean that our results likely underestimate the true burden of radiation-associated health problems in the Narodichesky region. However, children from the villages near and from the town of Narodichi (in the highest exposure quintile) may have had more severely ill children attend the health screenings, potentially overestimating the real association in those areas. However, both of these effects would be slight because participation was high and did not change significantly across exposure quintiles.

Respiratory infections are common in children, especially in those with suppressed myelopoiesis, as defined by deficits in erythrocyte, lymphocyte, and thrombocyte counts (Kolpakov et al. 1992; Speer et al. 1982). Reduced numbers of white and red blood cells and thrombocytes indicated such a process in this cohort of children (Stepanova et al. 2008). Eventually the child recovers from the respiratory infection, but with suppressed myelopoiesis the child may become infected again, and this cycle of infection and recovery may occur repeatedly. The repeated cycles of respiratory infection may produce chronic airway inflammation, with chronic, recurrent airway obstruction, perhaps strongest in the small airways. Chronic airway inflammation may in turn result in airway remodeling, with the development of bronchial fibrosis and progressive airway obstruction. Chronic respiratory infections could also lead to bronchiectasis, which can also influence chronic airway obstruction. We found increasing evidence of chronic airway obstruction (FEV_1_/FVC%, PEF, MEF_25_, MEF_50_, MEF_75_, and small airway hyperreactivity) and restriction (FVC, restrictive airway defect) with increasing quintiles of soil ^137^Cs in the NCC children. This is consistent with previously reported data (Yablokov 2009b). Restrictive ventilatory impairment in children could be caused by decreased pulmonary surfactant, reduced lung growth for age, chronic obstruction, or fibrotic changes. Changes in the fatty acid composition of pulmonary surfactant after chronic radiation exposure have been reported (Kolpakov 1999; Parkhomenko et al. 2008). Such changes in pulmonary surfactant could lead to restrictive ventilatory impairment. Survivors of childhood cancer who received radiation therapy have been reported to develop significant pulmonary problems, including lung fibrosis (Abid et al. 2001; Mertens et al. 2002). Radiation-induced pulmonary fibrosis will typically produce restrictive ventilatory impairment. Thus, the 2.60 times greater likelihood of restrictive ventilatory impairment observed in this study is plausible in light of the current medical literature.

Our findings provide evidence for three potential mechanisms that could cause the obstructive and restrictive spirometry results observed in the 415 children in this study who had pulmonary function testing. First of all, repeated exposure to gamma radiation, such as resulting from the decay of ^137^Cs, may cause immunosuppression (Carreno et al. 2002; Hamilton et al. 1986 Sen Gupta et al. 1979), which is likely to increase the susceptibility of children to develop respiratory infections. Repeated respiratory infections during lung development could retard lung growth, cause frequent episodes of inflammation-associated airway obstruction, cause airway remodeling with the subsequent development of airway fibrosis, or influence the development of bronchiectasis. Collectively, these mechanisms could lead to the observed reductions in PEF, FEV_1_/FVC%, MEF_25_, MEF_50_, and MEF_75_, indicative of airway obstruction. On the other hand, the development of restrictive ventilatory impairment could result from radiation-induced changes in the fatty acid composition of pulmonary surfactant and direct toxicity to lung tissue. Significant lung tissue injury after exposure to ^137^Cs could contribute to the subsequent development of lung fibrosis in affected children. It is possible that some children develop both airway obstruction and restrictive ventilatory impairment. Further studies with spirometry and lung volumes are needed to determine the extent of airway obstruction and restrictive ventilatory impairment among ^137^Cs exposed children. Additional studies with diffusion capacity for carbon monoxide would be useful in assessing the development of pulmonary fibrosis in exposed children.

## Conclusions

Thousands of peasant children are living in and consuming locally grown foods from areas where the soil is still profoundly contaminated with ^137^Cs (Cardis 2007). Hundreds of those children may grow up with lungs that are damaged from chronic exposure to the ^137^Cs radioisotope. The long-term prognosis of these children is poor. Some will probably develop significant respiratory problems as they age. The results of this study point to the need for further public health surveillance, continued environmental remediation, dietary intervention, and better risk communication among affected populations. Further studies are needed to elucidate the full spectrum of respiratory problems and other health consequences among children who have been chronically exposed to ^137^Cs after the Chernobyl accident.

## Figures and Tables

**Table 1:** Study population characteristics: Narodichi Children’s Cohort (NCC), Narodichesky, Ukraine, 1993–1998.

Characteristic	*n*	Percent
Sex: male	923	48.9
Age (years)		
2	2	0.1
3	26	1.4
4	82	4.3
5	142	7.5
6	138	7.3
7	164	8.7
8	225	11.9
9	225	11.9
10	205	10.9
11	177	9.4
12	187	9.9
13	145	7.7
14	93	4.9
15	48	2.5
16	28	1.5
17	1	0.1
Year of birth		
1980	111	5.9
1981	126	6.7
1982	150	7.9
1983	153	8.1
1984	312	16.5
1985	266	14.1
1986^a^		
1987	358	19.0
1988	293	15.5
1989	92	4.9
1990	24	1.3
1991	3	0.2
Year of participation		
1993	373	19.8
1994	359	19.0
1995	293	15.5
1996	337	17.8
1997	294	15.6
1998	232	12.3
> 1998	1,118	59.2
Mean ^137^Cs exposure quintile (kBq/m^2^)		
5: 355.47 ± 6.84	889	45.06
4: 308.63 ± 7.78	136	7.71
3: 195.45 ± 30.13	260	14.46
2: 128.2 ± 6.35	308	17.11
1: 90.7 ± 16.75	295	15.66

Final sample size: *n* = 1,888 measurements, 415 children.

a Data are being analyzed separately.

**Table 2:** Top 15 most commonly reported ICD-9 diagnosis codes: Narodichi Children’s Cohort (NCC), Narodichesky, Ukraine, 1993–1998 (*n* = 5,519).

ICD-9 code	Disease	Frequency	Percent
240.9	Goiter—unspecified	2,421	43.87
521, 521.0	Dental caries	2,140	38.78
575.1, 575.5, 575.8	Other cholecystitis	1,712	31.02
474, 474.0, 474.1, 474.2	Chronic disease of tonsils and adenoids	1,173	21.25
780.7	Malaise and fatigue	1,171	21.22
785.6	Enlargement of lymph nodes	1,091	19.77
289.1	Chronic lymphadenitis	1,004	18.19
576.1	Cholangitis	1,004	18.19
281.9	Other deficiency anemias—unspecified	813	14.73
783.4	Lack of expected normal physiologic development	533	9.66
337.9	Disorders of the autonomic nervous system—unspecified	515	9.33
535.5	Unspecified gastritis and gastroduodenitis	514	9.31
553.1	Umbilical hernia	417	7.56
737.9	Curvature of spine—unspecified	402	7.28
460	Acute nasopharyngitis (common cold)	358	6.49

**Table 3:** Multivariate mixed-effects regression models of percent predicted spirometry measures per quintile increase in soil ^137^Cs, Narodichi Children’s Cohort (NCC), Narodichesky, Ukraine, 1993–1998

Measure, mean ^137^Cs quintile (kBq/m^2^)	Adjusted mean	95% CI
FVC		
5: 355.5	84.47	82.65–86.30
4: 308.6	84.01	79.76–88.27
3: 195.5	93.31	90.22–96.40
2: 128.2	88.63	85.77–91.49
1: 90.7	90.82	87.84–93.80
FEV_1_/FVC%		
5: 355.5	95.51	94.02–97.00
4: 308.6	91.44	87.97–94.92
3: 195.5	96.12	93.59–98.64
2: 128.2	97.93	95.60–100.27
1: 90.7	99.66	97.23–102.10
PEF		
5: 355.5	81.47	79.45–83.49
4: 308.6	73.43	68.73–78.13
3: 195.5	83.64	80.22–87.05
2: 128.2	85.66	82.50–88.81
1: 90.7	85.56	82.27–88.86
MEF_25_		
5: 355.5	81.60	79.28–83.92
4: 3: 308.6	73.60	68.25–78.95
3: 95.5	83.80	79.93–87.67
2: 128.2	88.20	84.62–91.77
1: 90.7	89.24	85.49–92.98
MEF_50_		
5: 355.5	84.19	81.50–86.89
4: 308.6	76.13	69.84–82.42
3: 195.5	83.23	78.67–87.80
2: 128.2	91.10	86.88–95.33
1: 90.7	88.93	84.52–93.34
MEF_75_		
5: 355.5	78.31	74.89–81.73
4: 308.6	70.09	62.12–78.07
3: 195.5	78.91	73.12–84.69
2: 128.2	89.69	84.34–95.04
1: 90.7	89.15	83.57–94.74

Final sample size: *n* = 1,888 measurements, 415 children.

Quintiles 5–1 are presented for each measure.

**Table 4:** Multivariate mixed-effects logistic regression models of percent predicted spirometry measures per quintile increase in soil ^137^Cs: Narodichi Children’s Cohort (NCC), Narodichesky, Ukraine, 1993–1998

Measure, mean ^137^Cs quintiles (kBq/m^2^)	OR (95% CI)
FVC	
5: 355.5	2.60 (1.07–6.34)
4: 308.6	2.12 (0.62–7.21)
3: 195.5	0.44 (0.10–1.88)
2: 128.2	0.56 (0.16–1.97)
1: 90.7	1.00

FEV_1_/FVC%	
5: 355.5	5.08 (1.02–25.19)
4: 308.6	5.21 (0.85–32.03)
3: 195.5	2.66 (0.43–16.56)
2: 128.2	1.63 (0.25–10.77)
1: 90.7	1.00

PEF	
5: 355.5	1.61 (0.77–3.36)
4: 308.6	6.07 (2.09–17.62)
3: 195.5	0.72 (0.27–1.94)
2: 128.2	0.68 (0.27–1.72)
1: 90.7	1.00

Restrictive airway defect	
5: 355.5	2.26 (0.87–5.87)
4: 308.6	1.16 (0.26–5.15)
3: 195.5	0.48 (0.12–1.99)
2: 128.2	0.53 (0.14–1.95)
1: 90.7	1.00

Airway hyperreactivity	
Small airway	
5: 355.5	2.10 (0.80–5.50)
4: 308.6	5.83 (1.75–19.43)
3: 195.5	2.05 (0.65–6.50)
2: 128.2	1.41 (0.47–4.28)
1: 90.7	1.00

Middle airway	
5: 355.5	0.93 (0.42–2.08)
4: 308.6	0.71 (0.22–2.35)
3: 195.5	1.25 (0.47–3.36)
2: 128.2	0.83 (0.33–2.10)
1: 90.7	1.00

Large airway	
5: 355.5	0.86 (0.40–1.87)
4: 308.6	1.08 (0.36–3.25)
3: 195.5	1.25 (0.48–3.26)
2: 128.2	0.39 (0.14–1.07)
1: 90.7	1.00

Any region	
5: 355.5	1.33 (0.66–2.68)
4: 308.6	2.19 (0.75–6.34)
3: 195.5	1.45 (0.60–3.55)
2: 128.2	0.72 (0.32–1.64)
1: 90.7	1.00

Final sample size: *n* = 1,888 measurements, 415 children. Quintiles 5–1 are presented for each measure. Decreased measures are defined as < 80% (of predicted value for PEF and FVC, of FVC for FEV_1_/FVC%); restrictive airway defect, as both FVC < 80% predicted and FEV_1_/FVC% > 90%; and airway hyperreactivity, as having a > 10% improvement in FEV_1_ after bronchodilator administration (salbutamol).
